# Beneficial effect of statins in patients receiving chronic hemodialysis following percutaneous coronary intervention: **A nationwide retrospective cohort study**

**DOI:** 10.1038/s41598-018-27941-w

**Published:** 2018-06-26

**Authors:** Sang Hoon Kim, Hye Yun Jeong, Dong Ho Yang, Jinkwon Kim, So-Young Lee

**Affiliations:** 1 Division of Cardiology, Department of internal medicine, CHA University, Seongnam, South Korea; 2 Division of Nephrology, Department of Internal Medicine, CHA University, Seongnam, South Korea; 3 Department of Neurology, CHA Bundang Medical Center, CHA University, Seongnam, South Korea

## Abstract

The cardiovascular diseases are the leading cause of mortality in end-stage renal disease (ESRD) patients. However, roles of statins are still controversial in dialysis-dependent ESRD patients regardless of having proven coronary artery occlusive disease. The aim of this study was to examine the benefit of statin following percutaneous coronary intervention (PCI) in ESRD patients who have proven coronary artery occlusive disease. This study was based on the National Health Insurance Service-National Sample Cohort in South Korea. We included 150 ESRD patients on chronic hemodialysis who underwent PCI with stenting between 2002 and 2013. The primary outcome was a composite of myocardial infarction, stroke, and all-cause mortality. Multivariate time-dependent Cox regression analysis were performed, and statin therapy after PCI was treated as a time-dependent variable. During 3.15 ± 2.71 (mean ± standard deviation) years of follow-up, there were 82 patients with primary outcome. The adjusted hazard ratio for statin use was 0.54 [0.33–0.90] compared to no statin use. This study showed that statin has significant benefit on reducing adverse events risk in dialysis-dependent ESRD patients after PCI.

## Introduction

Patients with end-stage renal disease (ESRD) have a poor prognosis, and their mortality rates are 6.7–8.5 fold greater than those of the general population^1^. Cardiovascular diseases are known as the leading cause of morbidity and mortality, accounting for almost 50 percent of deaths in the ESRD population^1,2^. ESRD patients commonly have coexisting cardiovascular risk factors, and emerging evidence show that kidney disease itself is a risk factor for atherosclerosis and cardiovascular events^2,3^. As a consequence, ESRD patients frequently undergo percutaneous coronary intervention (PCI) with stenting.

Statins are lipid-lowering agents and are widely prescribed for the primary and secondary prevention of cardiovascular events. The clinical benefit of statin use after PCI is well established by multiple clinical trials^4–7^. On the other hand, benefit of statin is still controversial in ESRD patients receiving dialysis^8–11^. It has recently been suggested that proprotein convertase subtilisin/kexin 9 (PCSK9) inhibitors, new therapeutic option for the treatment of dyslipidemia, may would be beneficial in reducing cardiovascular risk in chronic kidney disease (CKD)^12^. Clinical prognosis after PCI is proportional to kidney function, and relatively poor in ESRD patients^13^. Therefore, optimal preventive medications after PCI are crucial for the high-risk patients with ESRD. We hypothesized that statin therapy following PCI would have beneficial effects on ESRD patients, and the aim of this study was to examine this hypothesis by conducting a cohort study using the nationwide health insurance claim data.

## Results

**Figure 1:**
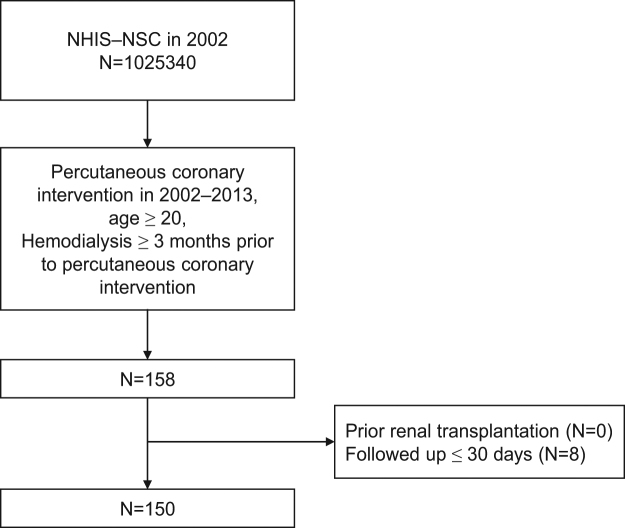
Flowchart of patient’s inclusion.

According to the inclusion and exclusion criteria, 150 chronic hemodialysis patients were finally included (Fig. 1). The median age at coronary artery implantation was 65–69 years old, and 56.7% was male (Table 1). During the first 30 days after PCI, 113 patients (75.3%) received statin therapy and 37 patients (24.7%) did not. Among 113 patients who received statin therapy, 80 patients were classified as good adherence (PDC ≥ 80%) and 33 patients were classified as poor adherence (PDC < 80%) during the first 30 days after PCI. Clinical characteristics were not significantly different between patient groups stratified by statin use during the first 30 days, except good adherence to aspirin and ADP receptor antagonist treatment, which was positively associated with good adherence to statin therapy (Table 1).

**Table 1:** Clinical characteristics of included patients according to the statin use during the first 30 days after percutaneous coronary intervention.

Variable	ALL, N = 150	no statin, N = 37	poor adherence (PDC < 80%), N = 33	good adherence (PDC ≥ 80%), N = 80	p-value^*^
Sex, male	85 (56.7)	20 (54.1)	20 (60.6)	45 (56.3)	0.853
Age	65–69 [60–94; 70–74]	65–69 [60–64; 70–74]	65–69 [60–94; 70–74]	65–69 [55–59; 70–74]	0.662
Hypertension	147 (98.0)	37 (100.0)	32 (96.97)	78 (97.5)	0.788
Diabetes mellitus	127 (84.7)	32 (86.5)	30 (90.9)	65 (81.3)	0.406
Atrial fibrillation	20 (13.3)	4 (10.8)	6 (18.2)	10 (12.5)	0.657
Acute myocardial infarction	51 (34.0)	8 (21.6)	14 (42.4)	29 (36.3)	0.153
Household income					0.942
Low tertile	46 (30.7)	10 (27.0)	10 (30.3)	26 (32.5)	
Middle tertile	49 (32.7)	14 (37.8)	11 (33.3)	24 (30.0)	
High tertile	55 (36.7)	13 (35.1)	12 (36.4)	30 (37.5)	
PDC by aspirin ≥0.8	116 (77.3)	26 (70.3)	18 (54.5)	72 (90.0)	<0.001
PDC by ADP receptor antagonist ≥0.8	115 (76.7)	21 (56.8)	21 (63.6)	73 (91.3)	<0.001

Data are number of patients (%) or median [interquartile range].

^*^Derived by chi square test for categorical data and the Kruskal-Wallis test for age between groups of no statin, poor adherence, and good adherence to statin.

ADP, Adenosine diphosphate receptor; PDC, proportion of days covered.

**Figure 2:**
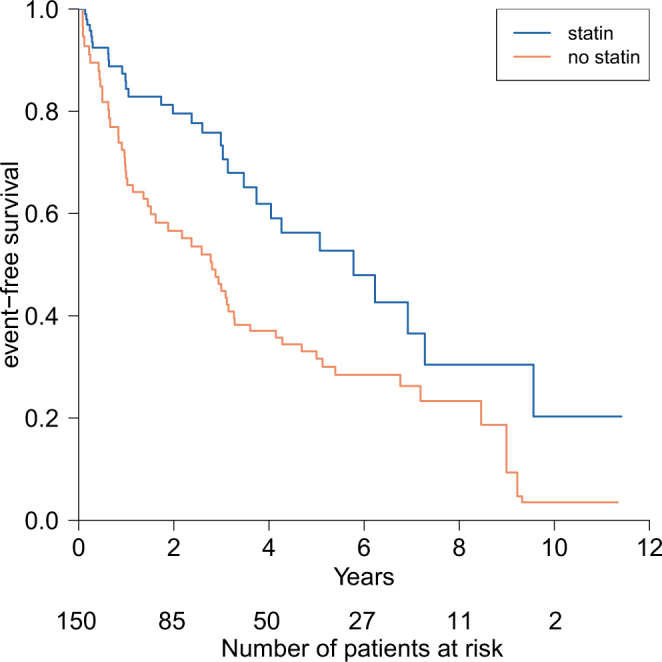
Simon and Makuch plot for event-free survival after coronary stent implantation in patients with end-stage renal disease by statin use.

During the follow-up period of 3.15 ± 2.71 (mean ± standard deviation) years, there were 82 patients with primary outcomes (17 cases of MI, 13 cases of stroke, and 52 cases of all-cause death by counting only the earliest event per patient). Figure 2 illustrates a survival plot based on the use of statin during the follow-up period. In the multivariate time-dependent Cox regression analyses, the adjusted HR [95% CI] for the use of statin was 0.54 [0.33–0.90] compared to no statin use (Table 2, Model A). Good adherence to statin (PDC_30day_ ≥ 0.8, PDC_fu_ ≥ 0.8) was also significantly associated with lower risk of adverse events (Table 2, Model B,C).

**Table 2:** Result of multivariate Cox regression models including statin use as time-dependent variable.

Cox models	Time dependent variable for statin therapy	Adjusted HR [95% CI]	p value
Model A	no use	reference	
	use	0.54 [0.33–0.90]	0.017
Model B	PDC_30day_ < 80%	reference	
	PDC_30day_ ≥ 80%	0.58 [0.35–0.95]	0.032
Model C	PDC_fu_ < 80%	reference	
	PDC_fu_ ≥ 80%	0.58 [0.35–0.96]	0.033

CI, confidence interval; HR, hazard ratio; PDC_30day_, proportion of days covered over the prior 30 days; PDC_fu_, proportion of days covered over the follow-up period from the index date to a given day.

Model A is adjusted for sex, age, the level of household income, the presence of hypertension, diabetes mellitus, atrial fibrillation, acute myocardial infarction as time-fixed covariates, and use of aspirin, and use of adenosine diphosphate receptor blocker as time-dependent covariates.

Model B is adjusted for same time-fixed covariates in the Model A, and PDC_30day_ by aspirin, and adenosine diphosphate receptor blocker ≥80% as time-dependent covariates.

Model C is adjusted for same time-fixed covariates in the Model A, and PDC_fu_ by aspirin, and adenosine diphosphate receptor blocker ≥80% as time-dependent covariates.

## Discussion

This study showed that statin therapy was significantly associated with reduced risk of adverse events in dialysis-dependent ESRD patients after PCI. This beneficial effect remained significant after adjusting for conventional risk factors and the use of antiplatelet agents. There was almost a 40% reduction in hazard for the primary outcome. Over the past decades, various effects of statins beyond lipid control have been reported in cardiovascular disease^14^. Statins play a role in improving vasomotor activity through promoting vasodilation, which is mediated by endothelial nitric oxide synthase^15,16^, and inhibiting vasoconstriction by the reduction of preproendothelin-1 transcription^17^. Statins are also immune modulators and impact various mechanisms, including inhibition of leukocyte adhesion to the endothelium, inhibition of inflammatory transcription factors, and reduction of serum inflammatory markers, such as C-reactive protein (CRP), tumor necrosis factor-α, and various cytokines and interleukins^18^. Indeed, several clinical trials have shown that statin therapy contributes to decreases in CRP levels^19–21^. In addition, inhibition of platelet aggregation and augmentation of thrombolysis have been suggested as other pleiotropic roles of statins^18,22–25^.

It has been demonstrated that CKD is associated with low-grade inflammation, endothelial dysfunction, and platelet activation, and thus, those alterations become worse as kidney function declines^26,27^. The Treating to New Targets (TNT) study on 3,107 patients with stable coronary artery disease and CKD found a significant benefit from high-dose atorvastatin (80 mg/day) on the reduction of coronary artery disease and stroke; however, patients who needed dialysis were not included^28^. The first large prospective trial of statin use in patients receiving chronic hemodialysis was conducted in Germany (the German Diabetes and Dialysis Study, 4D study)^8^. The results demonstrated that lowering LDL cholesterol with atorvastatin in 1,255 hemodialysis patients with type 2 diabetes but did not significantly reduce the incidence of primary outcomes (median 4 years)^8^. A few years later, the AURORA (A Study to Evaluate the Use of Rosuvastatin in Subjects on Regular Hemodialysis: An Assessment of Survival and Cardiovascular Events) Study Group examined the effects of rosuvastatin on patients undergoing regular hemodialysis treatment compared to a control group^9^. Rosuvastatin did not have a significant effect on the primary endpoint of nonfatal myocardial infarction, nonfatal stroke, or death from cardiovascular causes^9^. The recent large SHARP (Study of Heart and Renal Protection) trial studied a wide range of patients with CKD^11^. Although there was a significant risk reduction in primary outcomes among the non-dialysis-dependent patients with CKD after treatment with statins plus ezetimibe, dialysis patients did not benefit from the treatment^11^. The results of these three randomized controlled studies are inconsistent with prior large observational reports showing that statin prescriptions are associated with reduced mortality in chronic dialysis patients^29,30^.

In our study, we focused on chronic hemodialysis patients undergoing PCI because they are at high risk of cardiovascular disease, but there is no clear evidence regarding statin treatment among this patient population^31^. In both the 4D and SHARP studies, subjects who had recently undergone coronary intervention or experienced a myocardial infarction were excluded^8,11^. In the AURORA study, only 6.2% of subjects had a history of coronary revascularization before enrollment^9^. The European Society of Cardiology and European Atherosclerosis Society categorized patients with history of previous PCI as very high risk, and they recommended this group to reduce LDL-cholesterol level lower than 70 mg/dL. They recommended that dialysis-dependent CKD also must be considered very high risk, but they suggested not to initiate statin in case of dialysis-dependent CKD and free of atherosclerotic cardiovascular disease^32^. The United States Preventive Service Task Force recently emphasized statin therapy based on the presence of cardiovascular disease risk factors and a 10-year risk of cardiovascular disease events^33^. The American College of Cardiology and the American Heart Association also recommended high intensity statin therapy in CKD patients similar to the European group^34^. However, they did not make recommendation of statin use in the specific subgroup undergoing maintenance hemodialysis because of considerable uncertainty regarding whether statins have a cardiovascular protective effect on this population^8,9,11,34^. The Kidney Disease Improving Global Outcomes Lipid Guideline Development Work Group recommended that statins or the statin/ezetimibe combination should not be initiated in dialysis-dependent CKD patients without mentioning specific subgroups, such as patients who underwent coronary artery intervention^31^. In our retrospective cohort study, statin therapy has beneficial effects on dialysis-dependent patients following PCI. The selection of patients who underwent PCI in this study might have influenced the observation of a significant benefit from statin therapy compared to the prior trials with ESRD patients. Our results suggest that we may consider statin therapy in dialysis patients who underwent coronary intervention.

This study had both strengths and limitations aside from the retrospective nature. We were able to collect prescription data as well as admission and mortality data during the long-term follow-up period based on the national health claim data. In clinical practice, statin use is dynamic; the discontinuation and resumption of statin therapy are frequent and can occur at any time. Unlike previous observational studies, which commonly determined statin use at baseline or exposure during hospitalization^29,30^, we investigated statin use during the follow-up period as a time-dependent variable with consideration for the dynamics. This approach could strengthen the associations between time-varying covariates, including individual drug use, with clinical outcome compared to the traditional model^35^. On the other hand, we should acknowledge that outcome detection in this study might have limited accuracy. Although the diagnostic accuracy of the ICD-10 code in NHIS has been validated in prior studies, adverse events without admission could not be captured^36^. Additionally, we lacked information that was not available from the health insurance database, such as cigarette smoking status, biochemical abnormalities, the duration of dialysis, and why some patients get prescription of statin and others do not; these factors may cause confounding effects. Prescription data in the NHIS-NSC database did not assure the actual compliance of patients to medication. This study had retrospective observational design; therefore, we could not conclude the casual relationship between favorable prognosis and statin therapy. Additional study is required to establish the beneficial effects of adherent statin therapy on ESRD patients undergoing PCI.

## Methods

### Data source

This study used data from the National Health Insurance Service-National Sample Cohort (NHIS-NSC)^37^. All individuals living in South Korea are eligible for coverage from the National Health Insurance Program. The NHIS-NSC database was constructed with the health claim data of 1,025,340 subjects (2.2% of the total eligible Korean population in 2002) who were selected by stratified random sampling based on gender, age, and household income. The NHIS-NSC contains data of all health services, including hospital visits (inpatient and outpatient), medical procedures, prescriptions of drugs, diagnosis at the hospital visit, and demographic information, including sex, age, household income, and mortality, during the study period of 2002–2013. The diagnostic code was based on the International Classification of Diseases, 10th revision (ICD-10). There are many published articles based on the NIHS health claim data^38–41^.

### Study design

We conducted a retrospective cohort study using the NHIS-NSC database. The study subjects were patients on chronic hemodialysis who underwent PCI during 2002–2013. With the claim database of national health insurance program in Korea, we could find patients who underwent PCI with stenting (health claim code of ‘M6561’, ‘M6562’, ‘M6563’, and ‘M6564’) during the study period. Among them, we only included patients aged ≥20 years and who continued hemodialysis (health claim code of ‘O7020’ and ‘O9991’ in NHIS) for ≥3 months before PCI. As exclusion criteria, we excluded patients who underwent renal transplantation before PCI. Because too short follow-up period is inadequate to evaluate the adherence to statin, those followed-up <30 days were excluded. Figure 1 illustrates the inclusion and exclusion criteria. The index date was the first date of admission for PCI. Primary outcome was a composite of myocardial infarction (MI), stroke, and all-cause mortality, whichever occurred first after PCI. The development of MI (I21) and stroke (I60–I63) was defined as admission with a main diagnosis of the respective ICD-10 codes. In the validation studies based on the NHIS database, the diagnostic accuracy of MI was reported as 73–93%, and that for stroke was over 80%^27,42–44^. Patients were followed-up until the development of a primary outcome, renal transplantation, loss of eligibility for NHIS due to emigration, or Dec 31, 2013 (study end date). Data in the NHIS-NSC were fully anonymized and deidentified. This study was approved by the Institutional Review Board of CHA Bundang Medical Center, and informed consent was waived.

### Use of statins as a time-dependent variable

Starting from the index date, we collected the prescription records for statins (atorvastatin, rosuvastatin, pitavastatin, pravastatin, simvastatin, and lovastatin) in each patient. We determined the use of statin on each day of the follow-up period based on whether the day is covered by the prescription of statins. For each day, we calculated 1) the current use of statin on that day, 2) the proportion of days covered (PDC) by statin over the prior 30 days (PDC_30day_), and 3) the PDC by statin over the follow-up period from index date to a given day (PDC_fu_). Additional explanations of the definitions are shown in the Supplementary Table. PDC ≥80% is considered as a responsible cut-off value for the good adherence^45^. To reflect the dynamic change of statin use during follow-up, these values on each day were included as time-dependent variables in the survival analyses. Use of aspirin and adenosine diphosphate (ADP) receptor antagonists (clopidogrel, prasugrel, ticlopidine, and ticagrelor) were also assessed as time-dependent covariates in the same manner.

### Covariates as a time-fixed variable

We collected data on sex, age (grouped into five-year periods in the NHIS-NSC database), and household income (a marker of socioeconomic state at the time of PCI) as time-fixed covariates. The household income obtained from the NHIS-NSC was subdivided into tertile groups (Q1–Q3) for analysis. The presence of hypertension (I10–15), diabetes mellitus (DM; E08–11 and E13–14), and atrial fibrillation (AF; I48) was defined based on the presence of the diagnostic codes (ICD-10) in the NHIS-NSC before or at the time of discharge from the index admission. Hypertension and DM were recognized as relevant only if the subjects received anti-hypertensive (calcium-channel blockers, angiotensin-converting enzyme inhibitors, angiotensin-receptor blockers, diuretics, or beta-blockers) or anti-diabetic (sulfonylureas, biguanides, alpha-glucosidase inhibitors, thiazolidinediones, meglitinides, glucagon-like peptide-1 receptor agonists, dipeptidyl peptidase-4 inhibitors, or insulin) prescriptions along with the diagnostic code^46^. The presence of acute MI at PCI was determined by the presence of an ICD-10 code of I21 at the index admission.

### Statistical analysis

Comparison of clinical characteristics were performed using the chi square test for categorical data and the Kruskal-Wallis test for age. To evaluate effects of statin therapy on the risk of adverse events, we constructed three Cox proportional hazards regression models with fixed and time-varying covariates; each model included current use, PDC_30day_ ≥ 80%, and PDC_fu_ ≥ 80% as time-dependent variables for statin therapy on each day of the follow-up period. Adjustments were made for sex, age (as continuous variable), the level of house income, and the presence of hypertension, DM, atrial fibrillation, and acute MI as time-fixed covariates. Adjustments were also made for the use of antiplatelet drugs (aspirin or ADP receptor antagonist) as time-dependent covariates. The assumption of proportional hazards for statin therapy in the Cox models was tested by evaluating scaled Schonfeld residuals, which were found to be satisfactory. The survival plot with a time-dependent variable was illustrated using the method established by Simon and Makuch^47^. Data manipulation and the statistical analyses were performed with PostgreSQL, version 9.6.1 (The PostgreSQL Global Development Group; https://www.postgresql.org/) and R software, version 3.4.1 (The R Foundation for Statistical Computing, Vienna, Austria; http://www.R-project.org/). A two-sided P value of <0.05 was considered to be statistically significant.

## Electronic supplementary material

Supplementary Information
