# Supplementary material for: Beneficial effect of statins in patients receiving chronic hemodialysis following percutaneous coronary intervention: A nationwide retrospective cohort study
Source: Sci Rep. 2018 Jun 26;8:9692. doi: 10.1038/s41598-018-27941-w (PMC6018797; doi:10.1038/s41598-018-27941-w)
Supplement: Supplementary file 1 — Supplementary Information [file 41598_2018_27941_MOESM1_ESM.pdf]

Beneficial effect of statins in patients receiving chronic hemodialysis following percutaneous coronary intervention. A nationwide retrospective cohort study.

Sang Hoon Kim[1], Hye Yun Jeong[2], Dong Ho Yang[2], Jinkwon Kim[3], So-Young Lee[2]

[1]Division of Cardiology, [2]Division of Nephrology, Department of Internal Medicine, [3]Department of Neurology, CHA Bundang Medical Center, CHA University, Seongnam

Corresponding Author:

Dr. Jinkwon Kim, Department of Neurology, CHA University School of Medicine, CHA Bundang Medical Center, 59 Yatap-ro, Bundang-gu, Seongnam-si, 13496, South Korea. Phone: +82-31-780-5840, Fax: +82-31-780-5845, E-mail: antithrombus@gmail.com

Dr. So-Young Lee, Division of Nephrology, Department of Internal Medicine, CHA University School of Medicine, CHA Bundang Medical Center, 59 Yatap-ro, Bundang-gu, Seongnam-si, 13496, South Korea. Phone: +82-31-780-5025, Fax: +82-31-780-5219, E-mail: ysy0119@cha.ac.kr

S. H. Kim and H. Y. Jeong contributed equally to the work.

J. Kim and S. Lee contributed equally to the work

Supplementary Table. Example of time dependent variables for statin therapy in everyday of follow-up

person id	Time, day	statin prescription	time dependent variables for statin			outcome	comment
			use at the day	PDC_{30day}	PDC_{fu}		
1015	1		0		0/1	0	index date of admission for PCI
1015	2		0		0/2	0	
1015	3		0		0/3	0	
1015	4		0		0/4	0	
1015	5		0		0/5	0	
1015	6		0		0/6	0	
1015	7		0		0/7	0	
1015	8		0		0/8	0	
1015	9		0		0/9	0	
1015	10		0		0/10	0	
1015	11		0		0/11	0	
1015	12		0		0/12	0	
1015	13		0		0/13	0	
1015	14		0		0/14	0	
1015	15		0		0/15	0	
1015	16		0		0/16	0	
1015	17		0		0/17	0	
1015	18		0		0/18	0	
1015	19		0		0/19	0	
1015	20		0		0/20	0	
1015	21		0		0/21	0	
1015	22		0		0/22	0	
1015	23		0		0/23	0	
1015	24		0		0/24	0	
1015	25		0		0/25	0	
1015	26		0		0/26	0	
1015	27		0		0/27	0	
1015	28		0		0/28	0	
1015	29		0		0/29	0	
1015	30		0	0/30	0/30	0	
1015	31		0	0/30	0/31	0	
1015	32		0	0/30	0/32	0	
1015	33		0	0/30	0/33	0	
1015	34		0	0/30	0/34	0	
1015	35	atorvastatin x 10 days	1	1/30	1/35	0	
1015	36		1	2/30	2/36	0	
1015	37		1	3/30	3/37	0	
1015	38		1	4/30	4/38	0	
1015	39		1	5/30	5/39	0	
1015	40		1	6/30	6/40	0	
1015	41		1	7/30	7/41	0	
1015	42		1	8/30	8/42	0	
1015	43		1	9/30	9/43	0	
1015	44		1	10/30	10/44	0	
1015	45		0	10/30	10/45	0	
1015	46		0	10/30	10/46	0	
1015	47		0	10/30	10/47	0	
1015	48	atorvastatin x 20 days	1	11/30	11/48	0	
1015	49		1	12/30	12/49	0	
1015	50		1	13/30	13/50	0	
1015	51		1	14/30	14/51	0	
1015	52		1	15/30	15/52	0	
1015	53		1	16/30	16/53	0	
1015	54		1	17/30	17/54	0	
1015	55		1	18/30	18/55	0	
1015	56		1	19/30	19/56	0	
1015	57		1	20/30	20/57	0	
1015	58		1	21/30	21/58	0	
1015	59		1	22/30	22/59	0	
1015	60		1	23/30	23/60	0	
1015	61		1	24/30	24/61	0	
1015	62		1	25/30	25/62	0	

1015	63		1	26/30	26/63	0	
1015	64		1	27/30	27/64	0	
1015	65		1	27/30	28/65	0	
1015	66		1	27/30	29/66	0	
1015	67		1	27/30	30/67	0	
1015	68		0	26/30	30/68	0	
1015	69		0	25/30	30/69	0	
1015	70		0	24/30	30/70	1	primary outcome
2034	1	rosuvastatin x 20 days	1		1/1	0	
2034	2		1		2/2	0	
2034	3		1		3/3	0	
2034	4		1		4/4	0	
2034	5		1		5/5	0	
2034	6		1		6/6	0	
2034	7		1		7/7	0	
2034	8		1		8/8	0	
2034	9		1		9/9	0	
2034	10		1		10/10	0	
2034	11		1		11/11	0	
2034	12		1		12/12	0	
2034	13		1		13/13	0	
2034	14		1		14/14	0	
2034	15		1		15/15	0	
2034	16		1		16/16	0	
2034	17		1		17/17	0	
2034	18		1		18/18	0	
2034	19		1		19/19	0	
2034	20		1		20/20	0	
2034	21		0		20/21	0	
2034	22		0		20/22	0	
2034	23		0		20/23	0	
2034	24		0		20/24	0	
2034	25		0		20/25	0	
2034	26		0		20/26	0	
2034	27		0		20/27	0	
2034	28		0		20/28	0	
2034	29		0		20/29	0	
2034	30		0	20/30	20/30	0	
2034	31		0	19/30	20/31	0	
2034	32		0	18/30	20/32	0	
2034	33	rosuvastatin x 15 days	1	18/30	21/33	0	
2034	34		1	18/30	22/34	0	
2034	35		1	18/30	23/35	0	
2034	36		1	18/30	24/36	0	
2034	37		1	18/30	25/37	0	
2034	38		1	18/30	26/38	0	
2034	39		1	18/30	27/39	0	
2034	40		1	18/30	28/40	0	
2034	41		1	18/30	29/41	0	
2034	42		1	18/30	30/42	0	
2034	43		1	18/30	31/43	0	
2034	44		1	18/30	32/44	0	
2034	45		1	18/30	33/45	0	
2034	46		1	18/30	34/46	0	
2034	47		1	18/30	35/47	0	
2034	48		0	17/30	35/48	0	
2034	49		0	16/30	35/49	0	
2034	50		0	15/30	35/50	0	
2034	51		0	15/30	35/51	0	
2034	52		0	15/30	35/52	0	
2034	53		0	15/30	35/53	0	
2034	54		0	15/30	35/54	0	
2034	55		0	15/30	35/55	0	
2034	56	rosuvastatin x 7 days	1	16/30	36/56	0	
2034	57		1	17/30	37/57	0	

2034	58	1	18/30	38/58	0	
2034	59	1	19/30	39/59	0	
2034	60	1	20/30	40/60	0	
2034	61	1	21/30	41/61	0	
2034	62	1	22/30	42/62	0	
2034	63	0	21/30	42/63	0	
2034	64	0	20/30	42/64	0	
2034	65	0	19/30	42/65	0	
2034	66	0	18/30	42/66	0	
2034	67	0	17/30	42/67	0	
2034	68	0	16/30	42/68	0	
2034	69	0	15/30	42/69	0	
2034	70	0	14/30	42/70	0	
2034	71	0	13/30	42/71	0	
2034	72	0	12/30	42/72	0	
2034	73	0	11/30	42/73	0	
2034	74	0	10/30	42/74	0	
2034	75	0	9/30	42/75	0	
2034	76	0	8/30	42/76	0	
2034	77	0	7/30	42/77	0	
2034	78	0	6/30	42/78	0	
2034	79	0	5/30	42/79	0	censoring

PCI, percutaneous coronary intervention

PDC_{30day}, proportion of days covered over the prior 30 days

PDC_{fu}, proportion of days covered over the follow-up period from the index date to a given day
